# A Case of Cerebral Venous Sinus Thrombosis Following Eltrombopag in Immune Thrombocytopenia

**DOI:** 10.7759/cureus.96811

**Published:** 2025-11-13

**Authors:** Thayani Raja, Nicholsan Jesiah, Pakkiyaretnam Mayurathan, Aathithya Kugathasan

**Affiliations:** 1 University Medical Unit, Teaching Hospital Batticaloa, Batticaloa, LKA; 2 Clinical Sciences Department, Faculty of Health Care Sciences, Eastern University Sri Lanka, Batticaloa, LKA; 3 Intensive Care Unit, Teaching Hospital Batticaloa, Batticaloa, LKA

**Keywords:** cerebral venous sinus thrombosis, eltrombopag, immune thrombocytopenic purpura, low-molecular-weight heparin, thrombopoietin receptor agonist

## Abstract

Eltrombopag is a second-line treatment option for patients with immune thrombocytopenic purpura (ITP) who have persistent thrombocytopenia. Although generally considered safe, it has been associated with uncommon thromboembolic complications. We report an 18-year-old female with ITP who developed superior sagittal sinus thrombosis after starting eltrombopag. Eltrombopag was discontinued, and anticoagulation was initiated with subsequent clinical improvement. This case highlights a serious complication of eltrombopag therapy and underscores the need for vigilant monitoring of platelet counts, timely dose adjustment, and early recognition of thrombotic events in patients with ITP receiving thrombopoietin receptor agonists.

## Introduction

Immune thrombocytopenic purpura (ITP) is an autoimmune disorder marked by accelerated destruction of platelets in the periphery and reduced platelet formation from bone marrow megakaryocytes [1]. Patients often first present with bleeding symptoms caused by a severely reduced platelet count, most commonly petechiae, purpura, and mucosal bleeding. Fatal hemorrhage is more likely when the platelet counts drop under 30 × 10⁹ [2].

The goal of treatment is to prevent bleeding by maintaining adequate platelet counts. Corticosteroids, such as prednisolone, dexamethasone, and methylprednisolone, are first-line, while IVIG and anti-D are useful in emergencies but provide only transient platelet elevation [3]. Eltrombopag, a thrombopoietin receptor agonist (TPO-RA), is a second-line treatment choice for refractory thrombocytopenia in chronic ITP patients [4]. Although TPO-RAs are effective and generally safe, a major concern is thromboembolism, particularly at atypical sites such as cerebral venous sinus thrombosis (CVST).

We present a case of cerebral superior sagittal sinus thrombosis related to eltrombopag and discuss the importance of maintaining the desirable range of platelet counts in these patients.

## Case presentation

**Figure 1:**
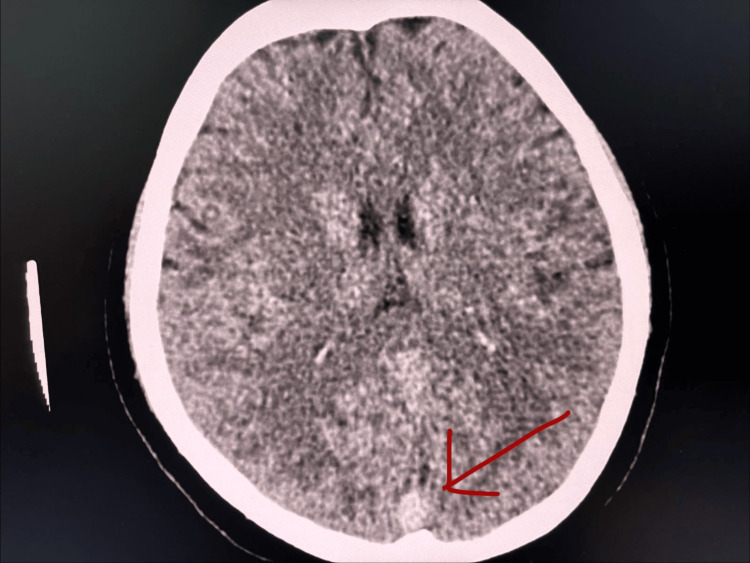
"Dense" delta sign in non-contrast CT brain

**Figure 2:**
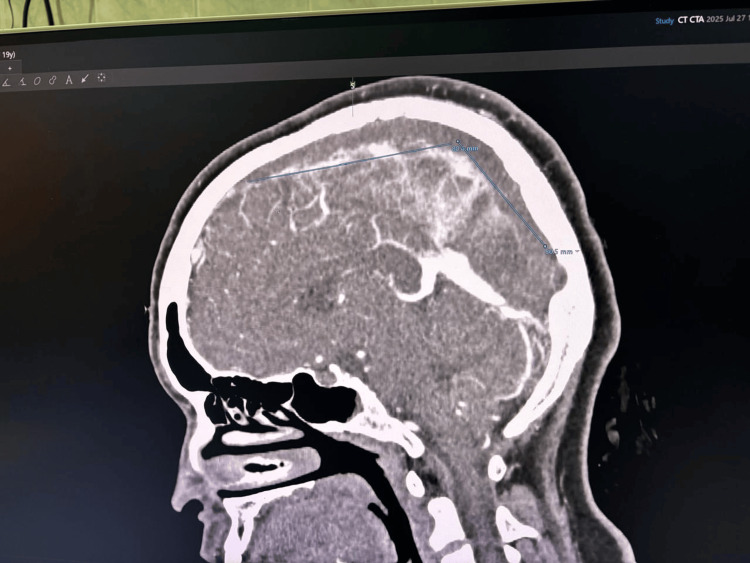
Extensive superior sagittal sinus thrombosis in CT venogram

An 18-year-old female with ITP presented with a headache for a three-day duration. She reported headache was associated with photophobia and phonophobia, and she vomited more than 10 times on admission. She did not complain about fever/limb weakness or fits/altered behavior/dizziness/weakness of limbs/abnormal movements. Her physical examination was unremarkable. Non-contrast CT brain showed "dense" delta sign suggestive of superior sagittal sinus thrombosis (Figure 1), and CT venogram confirmed superior sagittal sinus thrombosis extending to sigmoid sinus (Figure 2).

The patient has been diagnosed with ITP and was initially managed with glucocorticoids and intravenous immunoglobulin (IVIG), but the response was suboptimal. Approximately two weeks prior, eltrombopag 25 mg daily was initiated due to persistent thrombocytopenia and bleeding. Review of previous records revealed a platelet count of 8 × 10⁹/L before starting eltrombopag. On presentation, the platelet count was 123 × 10⁹/L and subsequently rose to 240 × 10⁹/L. Protein C, protein S, antithrombin, antiphospholipid antibodies, anti-nuclear antibody (ANA), rheumatoid factor, and hepatitis panels were negative, as were other autoimmune tests. Hence, a diagnosis of eltrombopag-associated CVST was made. The patient was discussed at a multidisciplinary team meeting involving a hematologist and a radiologist. Eltrombopag was discontinued, and the patient was commenced on low-molecular-weight heparin (LMWH) at 1 mg/kg subcutaneously every 12 hours. She was later transitioned to warfarin with heparin bridging to maintain a target INR of 2-3. Her clinical status improved to the extent that her headaches resolved. The patient was advised to follow up with a hematologist for close platelet monitoring, and warfarin was continued for a three-month duration.

## Discussion

Eltrombopag is an oral TPO-RA indicated for ITP refractory to steroids, immunosuppressants, or splenectomy, especially in patients at risk of bleeding [5]. It enhances platelet production by binding to the transmembrane domain of the thrombopoietin receptor (TPO-R), thereby stimulating the proliferation of megakaryocytes from bone marrow progenitor cells [6]. However, several studies have documented an elevated risk of both arterial and venous thrombosis with eltrombopag use [7,8]. Studies have described a spectrum of thrombotic complications associated with TPO-RA therapy, including pulmonary embolism, deep vein thrombosis, extensive CVST, portal vein thrombosis, ischemic stroke, and myocardial infarction [9,10].

Studies on eltrombopag and romiplostim have shown that thrombotic events may occur irrespective of platelet count, whether low, normal, or elevated [11,12]. In patients receiving eltrombopag, the target platelet count is generally maintained between 30-50× 10⁹/L to achieve an optimal balance between bleeding and thrombotic risk. Current recommendations suggest aiming for approximately 50× 10⁹/L, reducing the dose when counts exceed 150× 10⁹/L, and discontinuing therapy if platelet counts rise above 250 × 10⁹/L [13].

Reported management approaches in the literature commonly involved discontinuation of eltrombopag and initiation of anticoagulation therapy with either LMWH or warfarin, followed by gradual clinical recovery. This approach is consistent with the management of our patient, who demonstrated symptomatic improvement following cessation of eltrombopag and initiation of anticoagulation.

Overall, previously reported cases and the current observation indicate that thrombotic events associated with eltrombopag may occur early during therapy and across a range of platelet counts. The literature consistently emphasizes the need for close monitoring of platelet levels, adherence to target ranges, and awareness of early symptoms suggestive of thrombosis in patients receiving TPO-RAs.

## Conclusions

This case highlights the rare but serious complication of cerebral venous thrombosis in a patient with ITP receiving eltrombopag. While TPO-RAs remain an effective therapeutic option in refractory ITP, clinicians should remain vigilant for thrombotic complications, even at low or normal platelet counts. Careful patient selection, regular monitoring, and judicious dose adjustments are crucial to minimize risk.
